# Type III Secretion System of *Bradyrhizobium* sp. SUTN9-2 Obstructs Symbiosis with *Lotus* spp.

**DOI:** 10.1264/jsme2.ME20041

**Published:** 2020-07-02

**Authors:** Shun Hashimoto, Kohki Goto, Pongdet Pyromyou, Pongpan Songwattana, Teerana Greetatorn, Panlada Tittabutr, Nantakorn Boonkerd, Neung Teaumroong, Toshiki Uchiumi

**Affiliations:** 1 Graduate School of Science and Engineering, Kagoshima University, 1–21–35 Korimoto, Kagoshima, Kagoshima 890–0065, Japan; 2 School of Biotechnology, Institute of Agricultural Technology, Suranaree University of Technology, 111 University Avenue, Suranaree, Muang, Nakhon Ratchasima 30000, Thailand

**Keywords:** symbiosis, *Bradyrhizobium*, *Lotus* spp., type III secretion system, effector protein

## Abstract

The rhizobial type III secretion system secretes effector proteins into host plant cells, which may either promote or inhibit symbiosis with legumes. We herein demonstrated that the type III secretion system of *Bradyrhizobium* sp. SUTN9-2 obstructed symbiosis with *Lotus japonicus* Miyakojima, *L. japonicus* Gifu, and *Lotus burttii*. A mutant of SUTN9-2 that is unable to secrete effector proteins showed better nodulation and plant growth promotion than wild-type SUTN9-2 when paired with these *Lotus* spp. We propose that SUTN9-2 is a useful strain for understanding the mechanisms by which effector proteins obstruct symbiosis between *Bradyrhizobium* and *Lotus* spp.

Rhizobia induce the growth of symbiotic nitrogen-fixing organs, called nodules, on the roots of leguminous plants. Rhizobial nodulation factors (NFs) are key molecules for symbiosis (Lerouge *et al.*, 1990). NFs are lipochitooligosaccharides with a chitin oligomer backbone, the length and modifications of which are specific to rhizobial species (Ardourel *et al.*, 1994; Haeze and Holsters, 2002). After recognizing a compatible NF, the host legume activates nodulation signaling (Radutoiu *et al.*, 2003; Radutoiu *et al.*, 2007).

In addition to NFs, the rhizobial type III secretion system (T3SS) is an important factor for initiating symbiosis. Bacterial T3SS proteins, known as “nano syringes” or “injectisomes”, deliver effector proteins (type III effector proteins, T3Es) into target cells (Ryan and Stebbins, 2016). The T3SS of plant pathogenic bacteria, such as *Pseudomonas syringae*, suppress plant immunity and contribute to the virulence of the pathogen (Jakobek *et al.*, 1993). On the other hand, rhizobial T3SS may either promote or inhibit the establishment of symbiosis, depending on the host legume (Miwa and Okazaki, 2017). Rhizobial T3SS facilitate nodulation to promote symbiosis (Okazaki *et al.*, 2013), whereas T3SS trigger plant immune responses that suppress nodulation to inhibit symbiosis (Sugawara *et al.*, 2018; Kusakabe *et al.*, 2020).

*Bradyrhizobium* sp. SUTN9-2 was originally isolated from *Aeschynomene americana* nodules (Noisangiam *et al.*, 2012). SUTN9-2 has a wide host range and establishes symbiosis with legume species in several genera (Noisangiam *et al.*, 2012; Hashimoto *et al.*, 2019). To investigate the role of the T3SS of SUTN9-2 in symbiosis, a T3SS inactivation (Δ*T3SS*) mutant of SUTN9-2, which cannot deliver T3Es, was constructed by disrupting the *rhcJ* gene, which encodes a T3SS component (Piromyou *et al.*, 2015). Inactivation of the T3SS did not affect symbiosis of SUTN9-2 with the original host *A. americana*, which belongs to the Dalbergioids legume clade (Piromyou *et al.*, 2015). However, in symbiosis with *Vigna radiata* and *Macroptilium atropurpureum* (these plants belong to the Phaseolids legume clade), SUTN9-2Δ*T3SS* mutants induced a greater number of pink nodules and more effectively promoted plant growth than wild-type SUTN9-2 (Piromyou *et al.*, 2015). Thus, the T3SS of SUTN9-2 has a negative effect on symbiosis with *V. radiata* and *M. atropurpureum*. However, it currently remains unclear whether the T3SS of SUTN9-2 affects symbiosis with other host plants. In the present study, we focused on the symbiotic phenotypes of SUTN9-2 and its Δ*T3SS* mutant with a model legume of *Lotus japonicus* ecotypes B-129 Gifu and MG-20 Miyakojima as well as *Lotus burttii* B-303, which all belong to the Galegoids clade. We found that the T3SS of SUTN9-2 obstructed symbiosis with these three *Lotus* spp.

*Bradyrhizobium* sp. SUTN9-2 and its Δ*T3SS* mutant were grown at 28°C in modified yeast-mannitol medium (Giraud *et al.*, 2000). *Mesorhizobium loti* MAFF303099 (Kaneko *et al.*, 2000), an original microsymbiont of *L. japonicus*, was cultivated under the same conditions as SUTN9-2. *Lotus japonicus* Miyakojima MG-20 and Gifu B-129 and *L. burttii* B-303 were used as host plants.

The seeds of *Lotus* spp. were surface-sterilized in concentrated sulfuric acid for 10‍ ‍min followed by 0.2% sodium hypochlorite and 0.1% Tween 20 for 40‍ ‍min, and then washed with sterilized water. After surface sterilization, the seeds were transferred onto 0.8% agar plates and germinated at 28°C. Two-day-old seedlings were transferred to the top of a test tube containing vermiculite with buffered nodulation medium (Ehrhardt *et al.*, 1992) and grown at 28°C with a 12/12-h light/dark cycle. After 1‍ ‍week, each seedling was inoculated with 1‍ ‍mL of a rhizobial suspension adjusted to an OD_600_=1.0 with sterilized distilled water. Plant fresh weights, nodule numbers, and acetylene reduction activity (ARA; a marker of nitrogenase activity) were measured at 5 or 8‍ ‍weeks post-inoculation (wpi) according to Hashimoto *et al.* (2019).

When grown with *L. japonicus* Miyakojima, *Bradyrhizobium* sp. SUTN9-2 induced only white nodules with no ARA (Fig. 1B and E), and host plant growth was not promoted (Fig. 1A and C). However, SUTN9-2Δ*T3SS* induced pink nodules with ARA (Fig. 1B and E) and promoted host plant growth (Fig. 1A and C). The number of white nodules induced by SUTN9-2Δ*T3SS* was significantly lower than that induced by SUTN9-2 (Fig. 1D).

When *L. japonicus* Gifu was used as the host plant (Fig. 2), SUTN9-2 induced both white and pink nodules (65 and 35%, respectively, Fig. 2B and D). SUTN9-2Δ*T3SS* also induced white and pink nodules; however, the ratio of pink to white nodules was higher (pink, 74%; white, 26%) than that induced by SUTN9-2 (Fig. 2D). In addition, the number of white nodules induced by SUTN9-2Δ*T3SS* was significantly lower than that by SUTN9-2 (Fig. 2D). Plants inoculated with SUTN9-2Δ*T3SS* showed significantly better growth and 2.7-fold stronger ARA than those inoculated with SUTN9-2 (Fig. 2A, C, and E).

When *L. burttii* was used as the host (Fig. 3), SUTN9-2 induced both white and pink nodules (93 and 7%, respectively; Fig. 3B and D). On the other hand, SUTN9-2Δ*T3SS* induced 45% white and 55% pink nodules (Fig. 3B and D). The inoculation with SUTN9-2Δ*T3SS* produced significantly fewer white nodules and significantly more pink nodules than that with SUTN9-2 (Fig. 3D). Plants inoculated with SUTN9-2Δ*T3SS* showed significantly better growth and stronger ARA than those inoculated with SUTN9-2 (Fig. 3A, C, and E).

A previous study reported that the T3SS of *Bradyrhizobium* sp. SUTN9-2 negatively affected symbiosis with *V. radiata* and *M. atropurpureum*, but not symbiosis with the original host *A. americana* (Piromyou *et al.*, 2015). In the present study, we also found that the T3SS of SUTN9-2 obstructed symbiosis with *Lotus* spp. The inoculation with wild-type SUTN9-2 induced only white nodules, whereas that with SUTN9-2Δ*T3SS* induced pink nodules and promoted the growth of *L. japonicus* Miyakojima. In symbiosis with *L. japonicus* Gifu and* L. burttii*, SUTN9-2 induced pink nodules; however, the number of nodules and degree of plant growth promotion were lower than those with SUTN9-2Δ*T3SS*. These results suggest that the T3E(s) of SUTN9-2 interfere with symbiosis with *Lotus* spp.

Kusakabe *et al.* (2020) examined symbiosis between *Bradyrhizobium elkanii* USDA61 and *Lotus* spp. A phylogenetic analysis among *Bradyrhizobium* strains showed that SUTN9-2 belonged to the same clade as *B. yuanmingense* isolated from *Lespedeza cuneata* (Zhu *et al.*, 2002) and *B. liaoningense* isolated from soybean (Xu *et al.*, 1995), but to a different clade than *B. elkanii* strains (Fig. S1). However, the present results were consistent with the findings reported by Kusakabe *et al.* (2020) (Fig. 4), suggesting that the *T3SS* of *Bradyrhizobium* obstructed symbiosis with *Lotus* spp..

NopM (Nodulation outer protein M) of USDA61 is a T3E that suppresses pink nodule formation on *L. japonicus* Miyakojima (Kusakabe *et al.*, 2020) (Fig. 4A). *M. loti*, a symbiont of *Lotus* spp., does not possess the *nopM* gene on its genome (Kusakabe *et al.*, 2020). Based on comparisons with genome sequence data available in the MicroScope database (https://mage.genoscope.cns.fr/microscope/home/index.php) (Vallenet *et al.*, 2020), SUTN9-2 possesses a putative *nopM* gene (the accession number in MicroScope is shown in Table. S1 as SUTN92_v1_640013), the product of which shows approximately 75% amino acid sequence identity with NopM (accession number in DDBJ, LC471585) of USDA61. The putative NopM of SUTN9-2 contained the same leucine-rich repeat (LRR) and ubiquitin ligase domain as NopM of USDA61 (Fig. S2). The *nopM* gene of SUTN9-2 may inhibit the formation of pink nodules on this plant; however, the T3E(s) of SUTN9-2 responsible have yet to be identified.

The *nopM* of USDA61 also suppressed the formation of pink nodules on *L. burttii* (Kusakabe *et al.*, 2020) (Fig. 4C). SUTN9-2 induced pink nodules on *L. burttii* despite the presence of a putative *nopM* (Fig. 3B and D), similar to the *nopM* disruption mutant of USDA61 on *L. burttii* (Kusakabe *et al.*, 2020) (Fig. 4C). Interestingly, the putative NopM protein of SUTN9-2 had a smaller number of LRR than that of the NopM protein in USDA61 (Fig. S2). LRR in proteins are generally involved in interactions with other molecules. These results suggest that the NopM proteins of these two strains either have different affinities for their targets or have different targets. The difference in the LRR-number of NopM may be related to the different phenotypes in their symbiosis with *L. burttii*.

A recent study by Kusakabe *et al.* (2020) suggested that not only NopM, but also other T3E(s) of USDA61 interfered with symbiosis with *L. burttii* (Fig. 4C). However, the T3E(s) responsible have yet to be identified. Similar to USDA61, the inactivation of T3SS in SUTN9-2 showed a better symbiotic phenotype than that of the wild-type strain (Fig. 4C). This result suggests that T3E(s) common to SUTN9-2 and USDA61, but not to *M. loti*, interfere with symbiosis with *L. burttii*.

SUTN9-2 induced pink nodules on *L. japonicus* Gifu, in contrast to USDA61 (Fig. 2B, D, and 4B). The NopF protein (accession number in DDBJ, LC471586) of USDA61 has been identified as a T3E that inhibits rhizobial infection and nodulation on *L. japonicus* Gifu (Kusakabe *et al.*, 2020). Based on comparisons with genome sequence data available in the MicroScope database (Vallenet *et al.*, 2020), SUTN9-2 does not possess a gene encoding NopF. The absence of NopF in SUTN9-2 may explain why SUTN9-2 exhibited a better nodulation ability than USDA61 on *L. japonicus* Gifu (Fig. 4B). However, the ability of the USDA61 *nopF* disruption mutant (Δ*nopF* in Fig. 4) to induce the formation of pink nodules was lower than that of wild-type SUTN9-2 (Fig. 4B). This result suggests that, in addition to NopF, USDA61 may possess specific T3E(s) that interfere with symbiosis with *L. japonicus* Gifu.

The Δ*T3SS* mutants derived from both SUTN9-2 and USDA61 more effectively promoted the growth of *Lotus* spp. than their respective wild-type strains, but not as well as the original microsymbiont *M. loti* (Fig. 1, 2, and 3; Kusakabe *et al.*, 2020). This result suggests that not only T3SS, but also unknown rhizobial factor(s) of SUTN9-2 and USDA61 obstruct symbiosis with *Lotus* spp. In addition, the functions and target molecules of these T3Es in *Lotus* spp. cells remain unknown. Comparisons of the sequences of these putative T3Es among SUTN9-2, USDA61, and *M. loti* may provide a more detailed understanding of the functions of T3E proteins in *Lotus* spp. cells. Further functional experiments will reveal the functions of T3E proteins in *Lotus* spp. cells. *Lotus* spp. used in the present study are useful lines for further investigations to identify the target of T3E in host plant cells. Thus, the present results will contribute to clarifying the mechanisms by which rhizobial T3Es inhibit *Bradyrhizobium*-*Lotus* symbiosis.

## Citation

Hashimoto, S., Goto, K., Pyromyou, P., Songwattana, P., Greetatorn, T., Tittabutr, P., et al. (2020) Type III Secretion System of *Bradyrhizobium* sp. SUTN9-2 Obstructs Symbiosis with *Lotus* spp.. *Microbes Environ ***35**: ME20041.

https://doi.org/10.1264/jsme2.ME20041

## Supplementary Material

Supplementary Material

## Figures and Tables

**Fig. 1.**
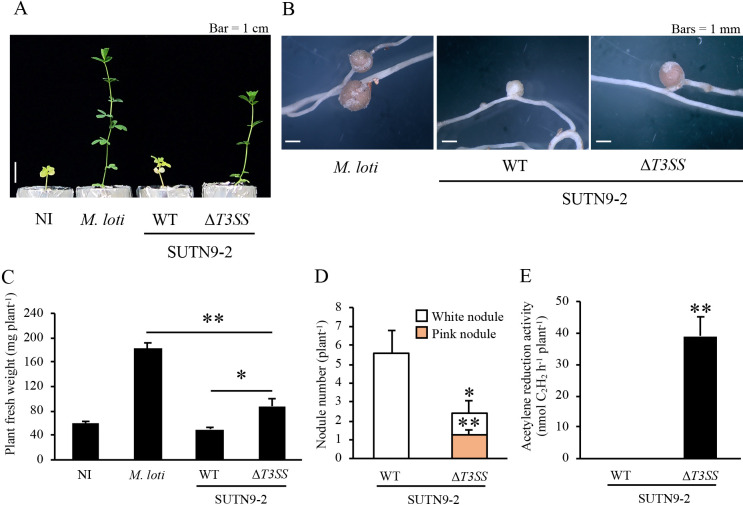
Symbiotic phenotypes of SUTN9-2 and SUTN9-2Δ*T3SS* with *Lotus japonicus* Miyakojima. All parameters were measured at 8 wpi. A, plant growth; B, nodules; C, plant fresh weight; D, nodule number; E, acetylene reduction activity. NI, no inoculum (control). WT, wild type. *Mesorhizobium loti* was used as a compatible strain. Values are means±SE (*n*=7), and asterisks indicate a significant difference (* *P*<0.05, ** *P*<0.01, the Student’s *t*-test).

**Fig. 2.**
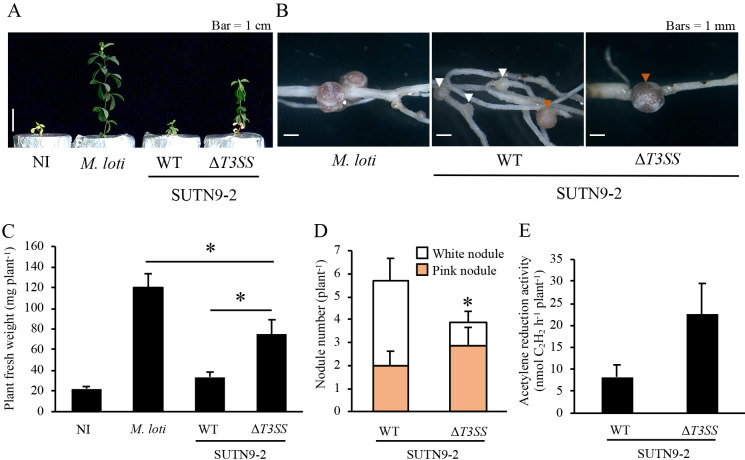
Symbiotic phenotypes of SUTN9-2 and SUTN9-2Δ*T3SS* with *Lotus japonicus* Gifu. All parameters were measured at 8 wpi. A, plant growth; B, nodules (white and orange arrowheads indicate white and pink nodules, respectively); C, plant fresh weight; D, nodule number; E, acetylene reduction activity. NI, no inoculum (control). WT, wild type. *Mesorhizobium loti* was used as a compatible strain. Values are means±SE (*n*=9), and asterisks indicate a significant difference (* *P*<0.05, the Student’s *t*-test).

**Fig. 3.**
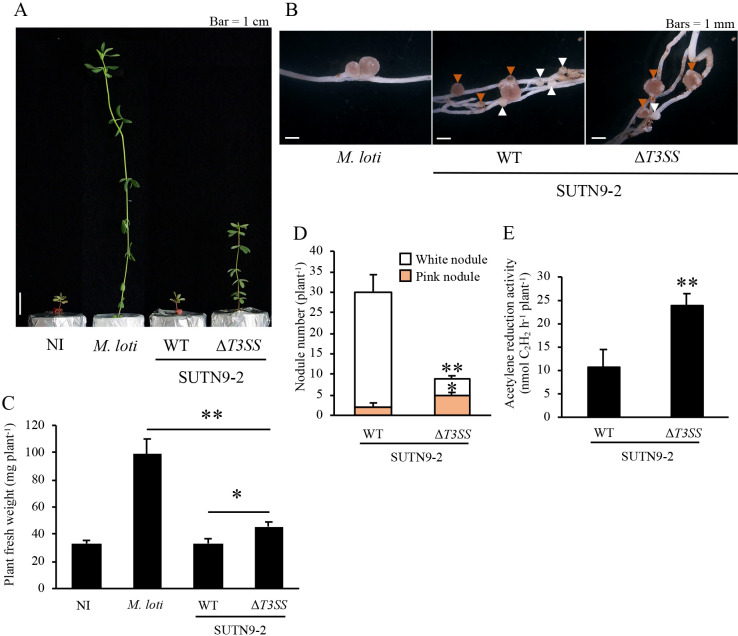
Symbiotic phenotypes of SUTN9-2 and SUTN9-2Δ*T3SS* with *Lotus burttii*. All parameters were measured at 5 wpi. A, plant growth; B, nodules (white and orange arrowheads indicate white and pink nodules, respectively); C, plant fresh weight; D, nodule number; E, acetylene reduction activity. NI, no inoculum (control). WT, wild type. *Mesorhizobium loti* was used as a compatible strain. Values are means±SE (*n*=11), and asterisks indicate a significant difference (* *P*<0.05, ** *P*<0.01, the Student’s *t*-test).

**Fig. 4.**
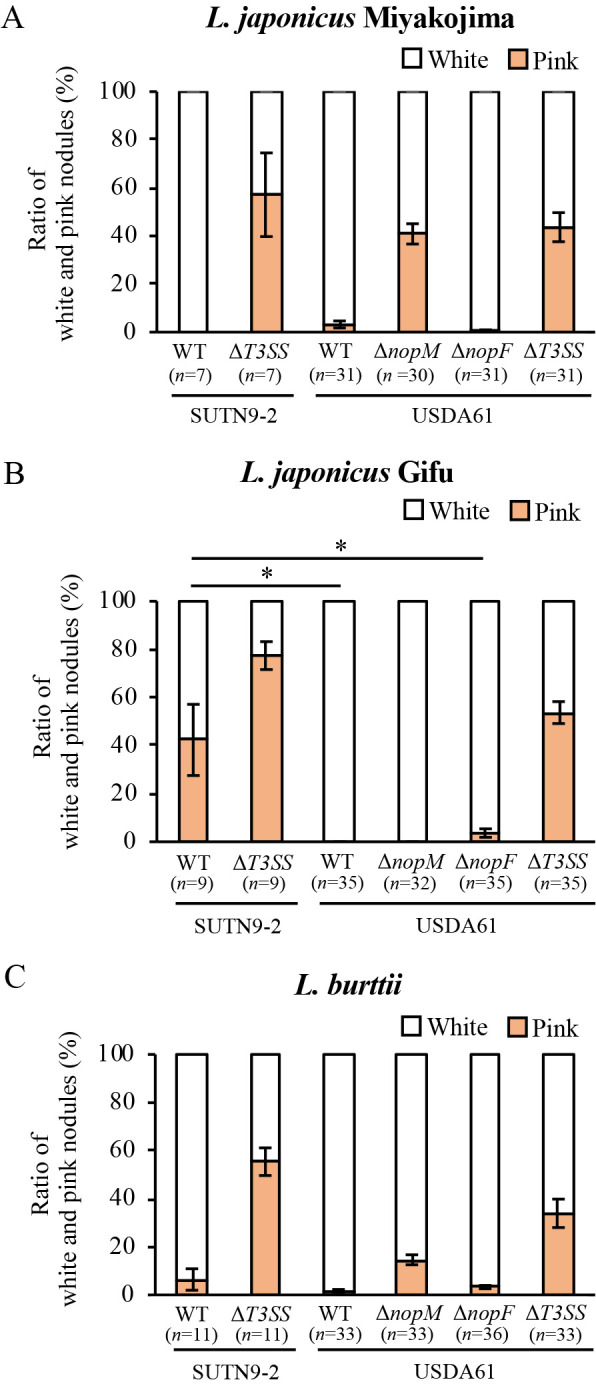
Comparison of the ratio of white and pink nodules induced by *Bradyrhizobium* sp. SUTN9-2, *Bradyrhizobium elkanii* USDA61, and their derivatives on *Lotus* spp. WT, wild type. The results for *B. elkanii* USDA61 and its derivatives were cited from Kusakabe *et al.* (2020). Values are means±SE, and asterisks indicate a significant difference (* *P*<0.05, the Student’s *t*-test).
