# Supplementary material for: Type III Secretion System of Bradyrhizobium sp. SUTN9-2 Obstructs Symbiosis with Lotus spp
Source: Microbes Environ. 2020 Jul 2;35(3):ME20041. doi: 10.1264/jsme2.ME20041 (PMC7511788; doi:10.1264/jsme2.ME20041)
Supplement: Supplementary file 1 — Supplementary Material [file 35_20041_s1.pdf]

Table S1. Accession number of sequences used in this study.

Strains	Sequence names	Labels in MicroScope	Accession numbers in DDBJ
Mesorhizobium loti MAFF303099	16S-rRNA	MAFFr03	—
Bradyrhizobium sp. SUTN9-2	16S-rRNA	SUTN92_v1_16s_rRNA_1	—
	Putative E3 ubiquitin-protein ligase (*nopM*)	SUTN92_v1_640013	—
B. diazoefficiens			
CCBAU41267	16S-rRNA	AJQI01_v1_rRNA1	—
NK6	16S-rRNA	NK6_4898	—
SEMIA5080	16S-rRNA	ADOU02_v1_rRNA3	—
USDA122	16S-rRNA	BD122_07305	—
Is-1	16S-rRNA	LGUJ01_v1_rRNA1	—
USDA110	16S-rRNA	Bjar01	—
B. elkanii			
587	16S-rRNA	AJJK01_v1_rRNA3	—
BLY3-8	16S-rRNA	LWUI01_v1_rRNA3	—
BLY6-1	16S-rRNA	LXEM01_v1_rRNA3	—
CCBAU05737	16S-rRNA	AJPV01_v1_rRNA3	—
CCBAU43297	16S-rRNA	AJPW01_v1_rRNA1	—
UASWS1015	16S-rRNA	JXOF01_v1_rRNA1	—
USDA3254	16S-rRNA	AXAH01_v1_rRNA3	—
USDA3259	16S-rRNA	AXAW01_v1_rRNA3	—
USDA61	16S-rRNA	—	AB231916
	Putative type III secretion system effector *nopF*	—	LC471586
	Putative type III secretion system effector *nopM*	—	LC471585
USDA76	16S-rRNA	ARAG_v1_16s_rRNA_1	—
USDA94	16S-rRNA	JAFC01_v1_rRNA3	—
WSM1741	16S-rRNA	AXAU01_v1_rRNA3	—
WSM2783	16S-rRNA	AXAP01_v1_rRNA9	—
B. japonicum			
22	16S-rRNA	AXVG01_v1_rRNA1	—
CCBAU83623	16S-rRNA	AJQB01_v1_rRNA2	—
CCBAU15354	16S-rRNA	AJPX01_v1_rRNA2	—
CCBAU15517	16S-rRNA	AJPY01_v1_rRNA2	—
CCBAU15618	16S-rRNA	AJPZ01_v1_rRNA1	—
CCBAU25435	16S-rRNA	AJQA01_v1_rRNA3	—
E109	16S-rRNA	RN69_07475	—
FN1	16S-rRNA	JGCL01_v1_rRNA3	—
in8p8	16S-rRNA	AUGD01_v1_rRNA3	—
Is-34	16S-rRNA	JRPN01_v1_rRNA1	—
is5	16S-rRNA	ATWP01_v1_rRNA1	—
J5	16S-rRNA	BKD09_RS07205	—
SEMIA5079	16S-rRNA	BJS_09120	—
USDA123	16S-rRNA	AXVP01_v1_rRNA3	—
USDA124	16S-rRNA	ARFJ_v1_16s_rRNA_1	—
USDA135	16S-rRNA	AXAT01_v1_rRNA3	—
USDA38	16S-rRNA	AXAG01_v1_rRNA5	—
USDA4	16S-rRNA	AXAF01_v1_rRNA3	—
USDA6	16S-rRNA	BJ6T_15140	—
B. liaoningense			
CCBAU05525	16S-rRNA	AJQC01_v1_rRNA1	—
CCBAU83689	16S-rRNA	AJQD01_v1_rRNA2	—
CCNWSX0360	16S-rRNA	LUKO01_v1_rRNA3	—
B. yuanmingense			
BR3267	16S-rRNA	LJYF_v1_rRNA3	—
CCBAU05623	16S-rRNA	AJQJ_v1_rRNA1	—
CCBAU10071	16S-rRNA	FMAE01_v1_rRNA3	—
CCBAU25021	16S-rRNA	AJQK01_v1_rRNA1	—
CCBAU35157	16S-rRNA	AJQL01_v1_rRNA1	—
P10 130	16S-rRNA	SATS_v1_rRNA1	—

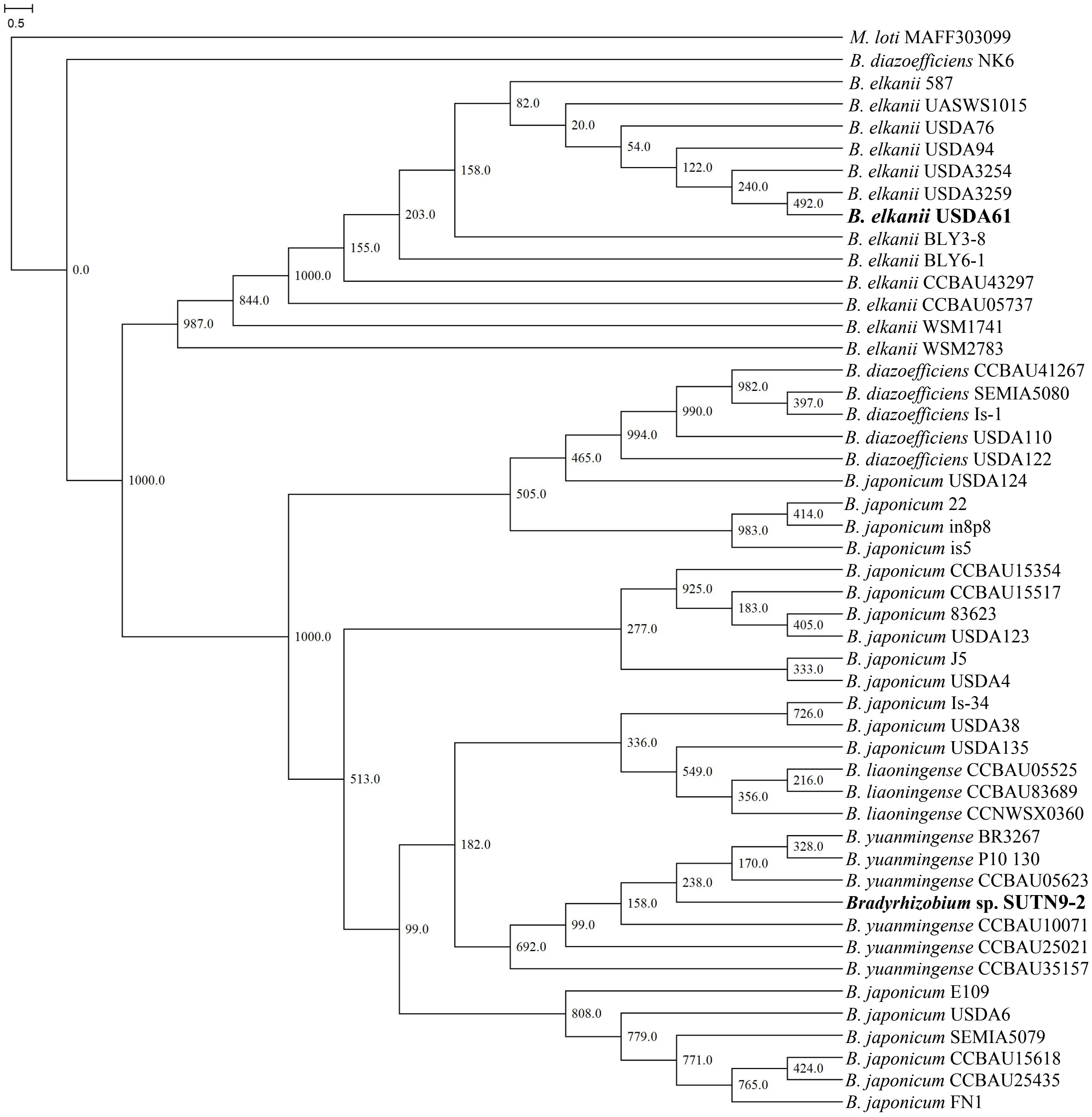

Fig. S1. Neighbor-joining phylogenetic tree of 16S-rRNA among *Bradyrhizobium* strains. Phylogenetic tree was generated from a ClustalW2 alignment, with 16S-rRNA of *Mesorhizobium loti* MAFF303099 as the outgroup. Bar shows five estimated substitutions of nucleic acid par 10 nucleic acid position. All accession number of sequences are listed in Table S1.

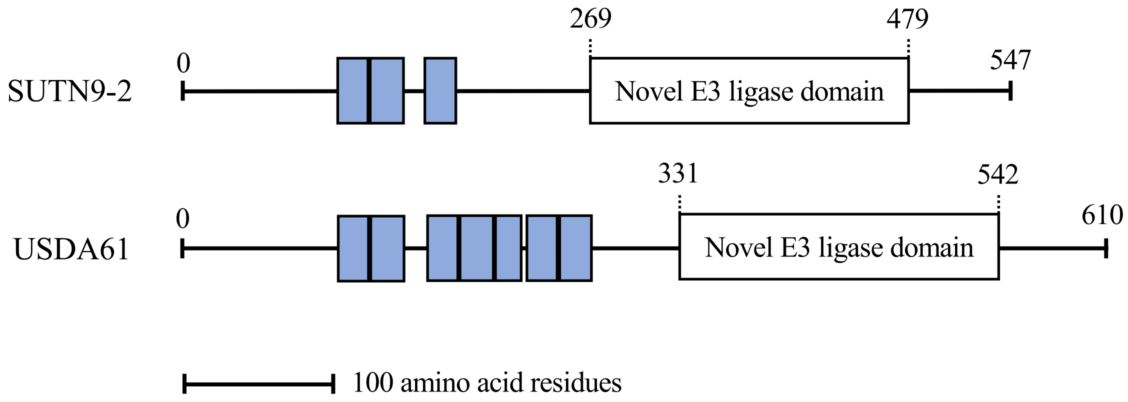

Fig. S2. Comparison of NopM proteins between *Bradyrhizobium* sp. SUTN9-2 and *B. elkanii* USDA61. Protein sequences were analyzed by Search Interpro (http://www.ebi.ac.uk/interpro/search/sequence/). Blue boxes, leucine rich repeats. In NopM of SUTN9-2, residues 103-123, 124-145 and 165-186 were presumed to be leucine rich repeat. In NopM of USDA61, residues 104-124, 125-146, 166-186, 187-208, 209-226, 228-248 and 249-270 were presumed to be leucine rich repeat.
